# Costunolide causes mitotic arrest and enhances radiosensitivity in human hepatocellular carcinoma cells

**DOI:** 10.1186/1748-717X-6-56

**Published:** 2011-05-30

**Authors:** Chia-Yuan Liu, Hsun-Shuo Chang, Ih-Sheng Chen, Chih-Jen Chen, Ming-Ling Hsu, Shu-Ling Fu, Yu-Jen Chen

**Affiliations:** 1 Institute of Traditional Medicine, National Yang-Ming University, Taipei, Taiwan; 2 Department of Medical Research, Mackay Memorial Hospital, Taipei, Taiwan; 3 Section of Gastroenterology, Department of Internal Medicine, Mackay Memorial Hospital, Taipei, Taiwan; 4 Department of Radiation Oncology, Mackay Memorial Hospital, Taipei, Taiwan; 5 Graduate Institute of Natural Products, College of Pharmacy, Kaohsiung Medical University, Kaohsiung, Taiwan

## Abstract

**Purpose:**

This work aimed to investigate the effect of costunolide, a sesquiterpene lactone isolated from *Michelia compressa*, on cell cycle distribution and radiosensitivity of human hepatocellular carcinoma (HCC) cells.

**Methods:**

The assessment used in this study included: cell viability assay, cell cycle analysis by DNA histogram, expression of phosphorylated histone H3 (Ser 10) by flow cytometer, mitotic index by Liu's stain and morphological observation, mitotic spindle alignment by immunofluorescence of alpha-tubulin, expression of cell cycle-related proteins by Western blotting, and radiation survival by clonogenic assay.

**Results:**

Our results show that costunolide reduced the viability of HA22T/VGH cells. It caused a rapid G2/M arrest at 4 hours shown by DNA histogram. The increase in phosphorylated histone H3 (Ser 10)-positive cells and mitotic index indicates costunolide-treated cells are arrested at mitosis, not G2, phase. Immunofluorescence of alpha-tubulin for spindle formation further demonstrated these cells are halted at metaphase. Costunolide up-regulated the expression of phosphorylated Chk2 (Thr 68), phosphorylated Cdc25c (Ser 216), phosphorylated Cdk1 (Tyr 15) and cyclin B1 in HA22T/VGH cells. At optimal condition causing mitotic arrest, costunolide sensitized HA22T/VGH HCC cells to ionizing radiation with sensitizer enhancement ratio up to 1.9.

**Conclusions:**

Costunolide could reduce the viability and arrest cell cycling at mitosis in hepatoma cells. Logical exploration of this mitosis-arresting activity for cancer therapeutics shows costunolide enhanced the killing effect of radiotherapy against human HCC cells.

## Background

Costunolide is a sesquiterpene lactone isolated from *Michelia compressa *in our previous work [1]. *Michelia compressa *is a common origin of wooden furniture used worldwide. Costunolide has been also identified in several species of plants, including Saussurea lappa C.B. Clarke [2], Aucklandia lappa Decne [3], Laurus nobilis [4], Magnolia grandiflora [5] and Michelia floribunda [6]. Bocca et al reported that costunolide interfered with the microtubule proteins [7]. However, whether this activity refers to mitosis arrest and subsequent applications for cancer therapy, such as radiosensitizing effect, remains unclear.

The primary liver cancers, in which 85 - 90% are hepatocellular carcinoma (HCC), is the third most common cause of death worldwide [8]. Despite aggressive therapy, the 5-year survival rate of patients with primary liver cancer remains less than 10% [9]. This poor prognosis is due to high recurrent and metastatic rates even after use of current treatment modalities such as surgery [10,11], trans-hepatic artery chemoembolzation (TACE) [12], radiofrequency ablation [13], radiotherapy (RT) [14], and multitarget tyrosine kinase inhibitors [15].

Among these treatment modalities, the role of RT, especially for unresectable HCC [16], is becoming important due to the development of advanced conformal techniques. The major organ at risk for irradiating hepatoma is the remaining normal liver containing normal hepatocytes. Although advanced conformal RT techniques could focus the radiation to hepatoma and reduce the dose to surrounding normal counterpart, the low tolerance of hepatocytes to radiation remains a limiting factor while attempting to escalate dose to the targeting tumor. Given that radiation dose is the only significant factor in predicting therapeutic effect of RT [17], development of novel radiosensitizers which would lower the necessary dose to eradicate hepatoma and thus cause less damages to normal liver is in great demand in clinical practice.

Because cells at G2/M phase, especially the M phase, are the most radiosensitive population, pharmacological agents possessing the microtubule-interfering activity have been shown as promising radiosensitizers. For example, taxane has been demonstrated as a radiosensitizer for treatment of non-small cell lung cancer [18,19]. Since costunolide has been reported as a microtubule-interfering agent by Bocca et al and shown to cause G2/M-arresting in our preliminary work, this compound may function as a radiosensitizer. To prove this working hypothesis, we examined whether costunolide induced cell cycle arrest specifically at G2 or M phase, investigated involved signaling pathways and measured the radiosensitivity of costunolide-treated hepatoma cells in this study.

## Methods

### Preparation of costunolide and determination of purity

Costunolide was isolated from root wood of *Michelia compressa *as previously described [1]. It was dissolved in dimethylsulfoxide (DMSO). Costunolide was stored as stock solution at -20° C. The working solution was freshly prepared prior to use. In all cell culture experiments, the final concentration of DMSO did not exceed 0.1% (v/v) which has no influence on the cell growth.

### Determination of drug purity

The samples were reconstituted with 100 mL methanol. The mobile phase was comprised of methanol and 10 mM sodium dihydrogen phosphate monohydrate in water (25:75, v/v, pH 6.5). The high performance liquid chromatography (HPLC) system was performed using a Shimadzu system (Shimadzu, Kyoto, Japan) consisting of a LC-20AT pump, a SIL-20AC auto-sampler, and an SPD-M20A detector. An Agilent extended-C18 column (4.6 × 150 mm, 5 μm) was used for separation (Merck, Germany). The UV absorbance at 204 nm wavelength was used for quantization. The retention time of costunolide was 6.31 minutes. Output data from the detector were integrated via a Class-VP 7.0 Client/Server Chromatography Data System (Shimadzu, Kyoto, Japan). Before subject to cell experiments, the purity of costunolide was examined. The optimum absorbance of costunolide is at 224 nm and the sample was inspected over a complete spectral range. Only one major peak can be seen in the HPLC analysis. According to the chromatogram, the purity of costunolide was approximately 99.9%.

### Cell culture and viability assessment

The poorly differentiated human HCC cell line, HA22T/VGH, was kindly provided by Professor Hu (Veteran General Hospital, Taipei, Taiwan). It was cultured in DMEM (GIBCO, Grand Island, NY, USA) supplemented with NaHCO_3 _(10 mmol/L), HEPES (20 mmol/L) and 10% heat-inactivated fetal calf serum (FCS, Hyclone, Logan, UT, USA) in a humidified 5% CO_2 _incubator and maintained in an exponential growth state. To evaluate cell growth, the numbers of viable cells were counted on day 1, 2 and 3 by using trypan blue exclusion test. Adherent cells were collected by using 0.25% trypsin.

### DNA histogram analysis

HA22T/VGH HCC cells were treated with 5 μM of costunolide for 0, 2, 4, 16 and 24 hours). Then the cells were harvested and washed with phosphate buffered saline (PBS), then fixed and permeated at 4°C for 1 hour with 70% ethanol. Cells were stained for 30 minutes with propidium iodide (PI) solution (PI, 0.5 mg/mL; RNAse, 0.1 mg/mL; Sigma) from a CycleTEST^plus ^DNA reagent kit (Becton Dickinson, Lincoln Park, NJ, USA) in the dark. Analysis of DNA histogram was performed on a FACScaliber flow cytometer (Becton Dickinson, Lincoln Park, NJ, USA). The data from 10^4 ^cells were collected and analyzed using ModFit Software (Becton Dickinson, Lincoln Park, NJ, USA) to calculate the percentage of cells at G2/M phase.

### Quantification of mitotic index

After treatment with 5 μM costunolide for 0, 4, 16 and 24 hours, HA22T/VGH HCC cells were collected and centrifuged onto a microscope slide using a Cytospin^2 ^centrifuge (Shandon Inc., Pittsburgh, PA, USA). The slides were dried and cells were fixed with 4% paraformaldehyde in PBS (pH 7.4) and mounted in Vectashield mounting medium with 1.5 Ag/mL 4V, 6-diamidino-2-phenylindole (Vector Laboratories, Inc., Burlingame, CA). The cells were stained by method of Liu's stain as follows: cells were washed by PBS and fixed by cold methanol for 20 min. Liu A was added for 45 seconds at room temperature followed by adding Liu B for 90 seconds. Then cells were gently washed and the cell morphology was observed by light microscope. Light micrograph was taken using a microscope (Olympus, Tokyo, Japan) at a magnification of 400 or 1000. Photograph was taken with a digital camera (Olympus, Tokyo, Japan). Mitotic morphology was identified by appearance of duplicated chromatid pair aligned in the center of dividing cells. At least 200 cells per field in a minimum of five randomly selected fields were counted on three slides for each sample.

### Detection of phosphorylated histone H3

The method for anti-phospho-histone H3 staining was performed and modified from a previous report [20]. In brief, growing cells were treated with 5 μM costunolide after 0, 2, 4, 16 and 24 hours. Then the HA22T/VGH HCC cells were trypsinized, fixed in 2% paraformaldehyd, permeablized with 1% Triton X-100 (Sigma), and stained with anti-phospho-histone 3 (Ser 10)-FITC (Cell Signaling, Danvers, MA, USA) at room temperature for 60 minutes. The cells were washed again with PBS and resuspended in PBS containing PI and RNase A. The samples were subjected to a FACScaliber flow cytometer and data analysis was done using CellQuest ^Pro ^software (Becton Dickinson, Lincoln Park, NJ, USA)

### Immunofluorescence staining

After 5 μM costunolide treatment, the HA22T/VGH cells were plated on a 18 mm coverslip coated with 50 mg/mL of Poly-*L*-Lysine. Cells were incubated at 37 °C to allow attachment and spreading. For immunofluorescence staining, the cells were fixed with 3% formaldehyde for 10 minutes. Then the cells were washed with PBS, permeabilized with 0.5% Triton X-100, stained with primary antibody (a-tubulin 1: 50, Zymed laboratories Inc., South San Francisco, CA) for one hour. After washing with PBS, the bound mouse IgG was detected with Cy™2-conjugated anti-mouse antibody (1: 100, Jackson ImmunoResearch, West Groove, PA) and counterstained with 0.5 mg/mL of Hoechst 33342 (Sigma) in PBS for one hour. Images of stained cells were examined under a fluorescent microscope (ZEISS, Axioplan 2).

### Western Blot analysis for expression of mitosis-related proteins

Cellular proteins were extracted, quantified, and subjected to gel electrophoresis. Protein samples were then blotted onto a polyvinylidene difluoride membrane. Primary antibodies against various proteins were used at various dilutions and detected by using horseradish peroxidase-conjugated anti-mouse immunoglobulin G followed by the use of enhanced chemiluminescence kits (Amersham Pharmacia Biotech). GAPDH expression was used as an internal control.

### Costunolide treatment and radiation delivery

Cells were plated onto culture dishes to allow grow in DMEM medium contained 10% FCS mixed with various concentrations of costunolide for 4 hours. Then costunolide was washed out and the cells were irradiated with various doses. Radiation therapy with 6 MeV electron beam energy was delivered by a linear accelerator (Clinac 1800, Varian Associates, Inc., Palo Alto, CA, USA) with dose rate 2.4 Gy/min at various dose (0, 0.5, 1, 2 and 3 Gy) in a single fraction. The selection of radiation doses depended on our preliminary work on calibration of radiation survival curves of HA22T/VGH cells to ensure adequate coverage from 100% to less than 37% survival (D_0 _in radiobiology) for further estimation of surviving fraction. To fit the clinical relevance, 2 Gy was also selected to match the daily fraction size commonly used in clinical practice. Full electron equilibrium was ensured for each fraction by a parallel plate PR-60C ionization chamber (CAPINTEL, Inc., Ramsey, NY, USA). After radiation, cells were plated for clonogenic assay.

### Clonogenic assay and estimation of SER

Viable tumor cells (10^3^) were plated into each 35-mm culture dish and allowed to grow in DMEM containing 10% FCS. After 10 -14 days, the culture dishes were stained with 3% crystal violet and the numbers of colony (more than 50 cells) were counted. The mean control plating efficiency for untreated HA22T/VGH HCC cells was around 43%. The surviving fraction was calculated as mean colonies/cells inoculated. Survival curves were fitted by a linear-quadratic model. The sensitizer enhancement ratio (SER) was calculated as the radiation dose needed for radiation alone divided by the dose needed for various concentrations of costunolide plus radiation at a survival fraction of 37% (D_0 _in radiobiology).

### Statistics

Data were presented as mean ± standard error from at least three experiments. IC_50 _values were calculated by GraphPad Prism 4 software (GraphPad Software, San Diego, California, USA). Statistical comparisons were made by using Student's *t*-test or one-way analysis of variance (ANOVA) as indicated. The difference was considered significant at *p *< 0.05. All data analysis was performed by using SPSS software (version 10.0, Chicago, IL, USA). We used Sigma Plot software (version 8.0, SPSS Inc., Chicago, IL, USA) with written syntax to fit survival curves with linear quadratic model.

## Results

### Cell viability and estimation of IC_50_

**Figure 1:**
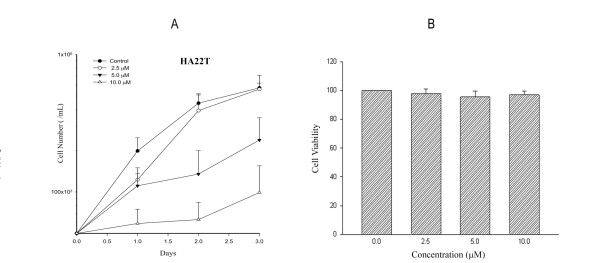
**Growth inhibition in hepatoma cells and human normal macrophages treated by costunolide**. Cell viability was assessed by trypan blue exclusion test for HA22T/VGH cells and MTT assay for macrophages. A, HA22T/VGH cells. B, Macrophages.

As demonstrated in Figure 1A, costunolide inhibited the viability of HA22T/VGH HCC cells in a concentration- and time-dependent manner. The estimated value of 50% inhibition concentration (IC_50_) was 4.7 μM. To sensitize tumor cell to radiation at a concentration range not extensively cytotoxic, costunolide at and below 5 μM was used for further cell cycle analysis and radiosensitizing experiments. Costunolide has no significant toxicity to normal human macrophages under the same experimental condition for HCC cells (Figure 1B).

### Cell cycle analysis by DNA histogram

**Figure 2:**
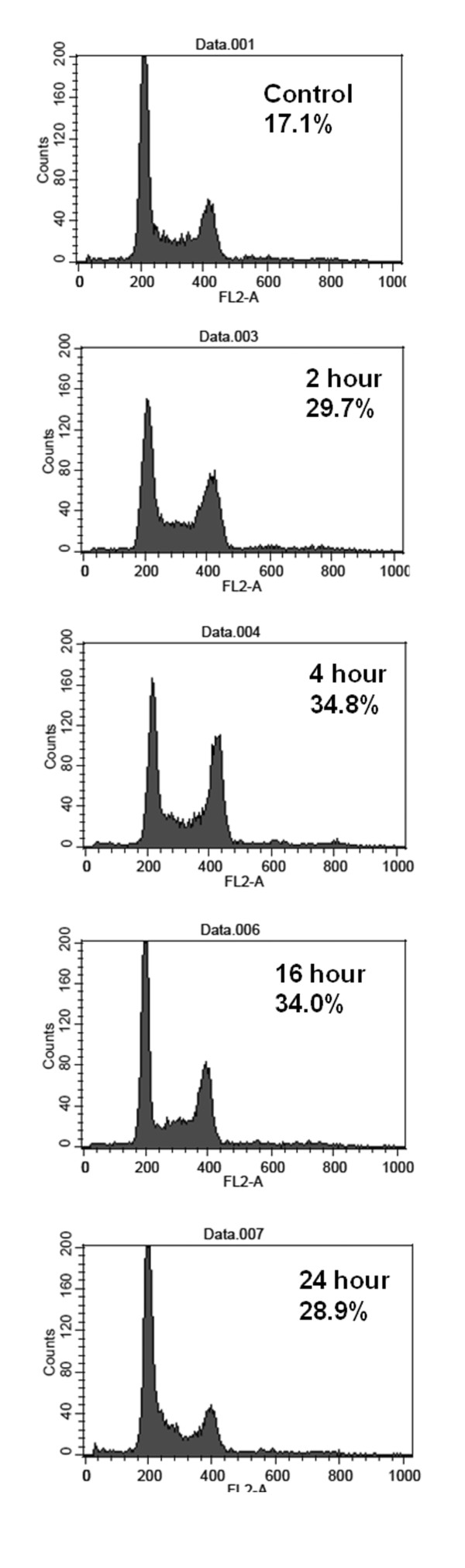
**Cell cycle analysis for HA22T/VGH cells treated by costunolide**. Cells were treated with costunolide (5 μM for various time periods as indicated). Representative DNA histograms were demonstrated.

After 5 μM costunolide treatment for 0, 2, 4, 16 and 24 hours, the percentage of G2/M increased up to a high level at 4 h (34.8 ± 0.5%), indicating a rapid G2/M arresting activity (Table 1, Figure 2). It was accompanied by slight incline of S phase and marked decline of G0/G1 phase (Table 1, Figure 2).

**Table 1:** The cell cycle distribution, phosphorylated H3-positive rates and mitotic index of HA22T/VGH cells after costunolide treatment

Costunolide 5 μM	Cell cycle (%)			histone H3 (%)	mitotic index(%)
			
	G1/G0	S	G2/M		
control	56.1 ± 0.7	26.8 ± 1.2	17.1 ± 1.5	3.6 ± 0.2	4.9 ± 0.7
2 h	33.0 ± 0.1	37.3 ± 0.3	29.7 ± 0.3	14.8 ± 2.6	17.4 ± 1.6
4 h	32.7 ± 0.7	33.1 ± 0.4	34.8 ± 0.5	25.8 ± 0.8	22.4 ± 4.1
16 h	39.3 ± 1.7	26.7 ± 4.7	34.0 ± 3.2	15.2 ± 1.8	7.8 ± 1.0
24 h	55.9 ± 2.1	15.2 ± 3.3	28.9 ± 1.2	8.2 ± 0.7	5.4 ± 0.7

### Discrimination of mitosis arrest, other than G2 phase

**Figure 3:**
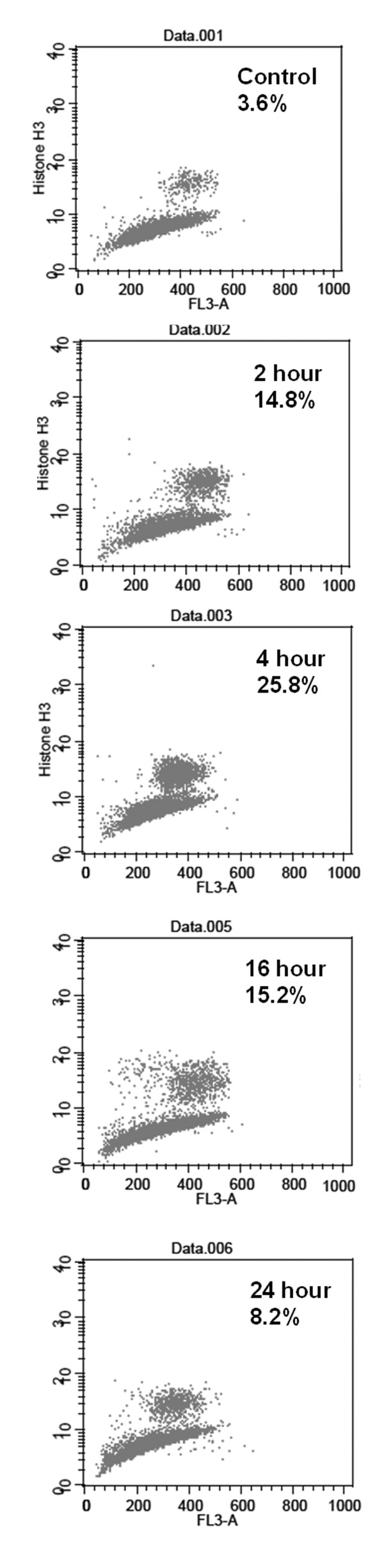
**Flow cytometric analysis for expression of phosphorylated histone H3 (Ser 10) in costunolide-treated HA22T/VGH cells**. Cells were treated with costunolide (5 μM for various time periods as indicated). Representative dot-plot visuals were demonstrated.

**Figure 4:**
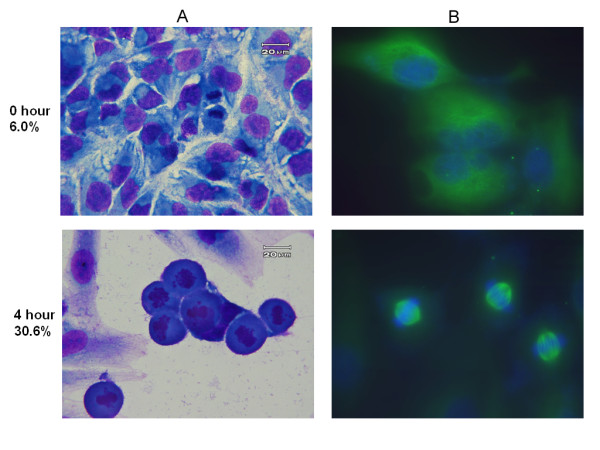
**Morphology of HA22T/VGH cells treated by 5 μM costunolide for 4 h**. A. Liu's stain; B. Immunofluorescence stain for alpha-tubulin. Magnification: ×1000.

To determine whether costunolide induced cell cycle arrest specifically at mitosis or G2 phase, we examined the phosphorylation status of histone H3 (Ser 10) and mitotic index, the hall markers of mitosis. By using nocodazole as a positive control (data not shown), the fluorocytometric assessment revealed a markedly corresponding increase in the percentage of phosphorylated histone H3-positive cells after costunolide treatment (Table 1, Figure 3). Mitotic index determined by morphological changes showed a similar pattern of changes (Table 1, Figure 4A). These results indicated that treatment with costunolide caused an arrest at mitosis, but not G2, phase in human hepatoma HA22T/VGH cells.

### Immunofluorescent stain for mitotic spindle

There are four stages in the mitotic phase. Immunofluorescent staining with alpha-tubulin was used to identify the cells located at which stage during the mitotic phase. In Figure 4B, the majority of the mitotic cells exhibited microtubule capture at both kinetochores of a duplicated chromatid pair which aligned in the center of the nucleus. The duplicated chromosome pairs were not separated apart and, instead, aggregated in the center of the nucleus in a round cell contour. These findings indicated a mitotic arrest at the metaphase was induced by costunolide.

### Signaling molecules associated with mitosis arrest

**Figure 5:**
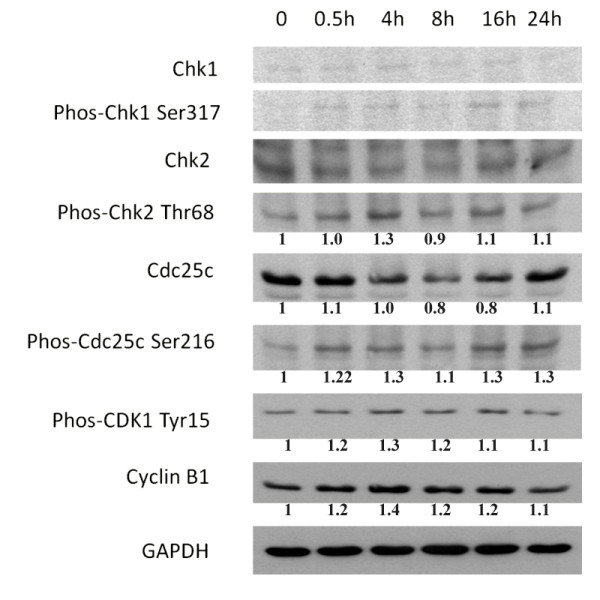
**The expression of mitosis arrest-related proteins after costunolide treatment in hepatoma cells**. Lane 1, untreated control; lane 2 - 6, treated with 5 μM costunolide for various time points.

As demonstrated in Figure 5, costunolide up-regulated the expression of phosphorylated Chk2 (Thr 68) up to 1.3 folds, phosphorylated Cdc25c (Ser 216) up to 1.3 folds, phosphorylated Cdk1 (Tyr 15) up to 1.3 folds and cyclin B1 up to 1.4 folds in HA22T/VGH cells. All these changes were greatest at 4 hours after costunolide treatment. No significant change was noted in the expression of phosphorylated Chk1 (Ser 317).

### Clonogenic survival and radiosensitization assessment

**Figure 6:**
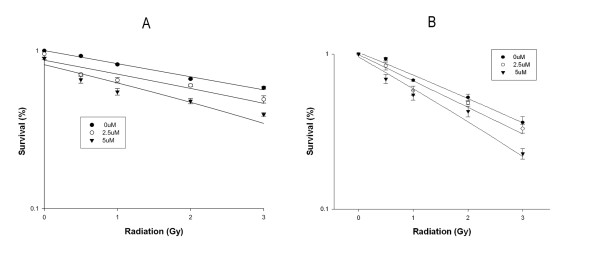
**Costunolide enhances the radiosensitivity of hepatoma cells**. A, HA22T/VGH cells. B, Sk-Hep1 cells. Clonogenic assay was used to estimate the survival of hepatoma cells.

At the effective condition causing mitotic arrest, costunolide at 2.5 and 5 μM sensitized HA22T/VGH HCC cells to ionizing radiation with SERs up to 1.3 and 1.9, respectively (Figure 6A). For another hepatoma cell line, costunolide inhibited the radiation survival of Sk-Hep1 cells in a way resembling HA22T cells. The SERs for Sk-Hep1 was up to 1.5 at 5 μM of costunolide (Figure 6B).

## Discussion

Several novel radiosensitizers have been isolated from natural products via various kinds of pathways. In comparison to paclitaxel, a known spindle poison with radiosensitizing activity, costunolide pretreatment resulted in a similar SER at a less toxic concentration to cells. The parthenolide enhanced the radiation sensitivity of p53 null PC-3 cells by a dose modification factor of 1.7 [21]. Caffeic acid phenethyl ester, isolated from bee propolis, possesses a SER of 2.2 for rectal adenocarcinoma CT26 cells [22]. In general, SER values around 2.0 are acceptable for development of radiosensitizers.

The relationship between cell cycle and radiosensitization effect [23] has been extensively investigated. Both G2 and M phases were identified as radiosensitive phases. Moreover, cells at M phase were proven to be more sensitive to radiation than those at G2 phase [24-26]. Based on the data from phosphorylation status of histone H3, mitotic index and alpha-tubulin immunofluorescence stain, we specified that costunolide caused mitotic arrest at or close to metaphase, but not G2 phase. This mitosis-arresting activity might be further referred to the radiosensitizing effect of costinolide on hepatoma cells as we demonstrated in the present study.

Costunolide up-regulated the expression of phosphorylated Chk2 (Thr 68), phosphorylated Cdc25c (Ser 216), phosphorylated Cdk1 (Tyr 15) and cyclin B1 in HA22T/VGH cells. It is known that activated Chk2 could prevent mitotic progression by phosphorylating Cdc25C at Ser216, enhancing Cdc25C-14-3-3 binding to sequester Cdc25C in the cytoplasm and preventing dephosphorylation of Cdk1 (Tyr 15 or Thr 14) to inhibit the mitotic progression [27]. Thus, this modulation of Chk2/Cdc25c/Cdk1/cyclin B1 signaling by costunolide may contribute to the mitotic arrest in HA22T/VGH cells.

Given that costunolide is a naturally occurring compound with great quantity in wood *Michelia compressa *and other plants, unravel of this novel bioactivity for radiosensitization may shed a light in development of new pharmaceutical agents from agricultural products by using this experimental model.

In conclusion, costunolide specifically arrests cell cycle at mitosis accompanied by modulation of Chk2/Cdc25c/Cdk1/cyclin B1 signaling and enhancement of radioresponse in human hepatoma HA22T/VGH cells. Further studies of its effect on both hepatoma and normal liver counterpart by experimental animal model should be performed before consideration in clinical trial.

## Competing interests

The authors declare that they have no competing interests.

## Authors' contributions

CYL participated in the design of the study and performed the cell cycle analysis and radiosensitivity experiment. HSC and ISC both purified chemical compound constunolide. CJC participated in its design and coordination of manuscript. MLH performed the expression of protein assay. YJC and SLF both conceived of the study, and participated in its design and coordination and helped to draft the manuscript. All authors read and approved the final manuscript.

## References

[B1] Liu CY Chen YW Cheng MJ Lee SJ Abd El-Razek MH Chang WH Chen YJ Chen IS Cytotoxic constituents from the root wood of formosan Michelia compressa J Chil Chem Soc 2008 53 1523 1524

[B2] Pandey MM Rastogi S Rawat AK Saussurea costus: botanical, chemical and pharmacological review of an ayurvedic medicinal plant J Ethnopharmacol 2007 110 379 390 10.1016/j.jep.2006.12.033 17306480

[B3] Li A Sun A Liu R Preparative isolation and purification of costunolide and dehydrocostuslactone from Aucklandia lappa Decne by high-speed counter-current chromatography J Chromatogr A 2005 1076 193 197 10.1016/j.chroma.2005.04.042 15974088

[B4] De Marino S Borbone N Zollo F Ianaro A Di Meglio P Iorizzi M New sesquiterpene lactones from Laurus nobilis leaves as inhibitors of nitric oxide production Planta Med 2005 71 706 710 10.1055/s-2005-864191 16142632

[B5] Koo TH Lee JH Park YJ Hong YS Kim HS Kim KW Lee JJ A sesquiterpene lactone, costunolide, from Magnolia grandiflora inhibits NF-kappa B by targeting I kappa B phosphorylation Planta Med 2001 67 103 107 10.1055/s-2001-11503 11301852

[B6] Mondranondra IO Che CT Rimando AM Vajrodaya S Fong HH Farnsworth NR Sesquiterpene lactones and other constituents from a cytotoxic extract of Michelia floribunda Pharm Res 1990 7 1269 1272 10.1023/A:1015937921880 2095565

[B7] Bocca C Gabriel L Bozzo F Miglietta A A sesquiterpene lactone, costunolide, interacts with microtubule protein and inhibits the growth of MCF-7 cells Chem Bio Interacti 2004 147 79 86 10.1016/j.cbi.2003.10.008 14726154

[B8] Jemal A Siegel R Ward E Hao Y Xu J Thun MJ Cancer statistics, 2009 CA Cancer J Clin 2009 59 225 249 10.3322/caac.20006 19474385

[B9] Hertl M Cosimi AB Liver transplantation for malignancy Oncologist 2005 10 269 281 10.1634/theoncologist.10-4-269 15821247

[B10] Poon RT Fan ST Lo CM Liu CL Wong J Intrahepatic recurrence after curative resection of hepatocellular carcinoma: long-term results of treatment and prognostic factors Ann Surg 1999 229 216 222 10.1097/00000658-199902000-00009 10024103 PMC1191634

[B11] Yamamoto J Kosuge T Takayama T Shimada K Yamasaki S Ozaki H Yamaguchi N Makuuchi M Recurrence of hepatocellular carcinoma after surgery Br J Surg 1996 83 1219 1222 10.1002/bjs.1800830913 8983610

[B12] Pelletier G Roche A Ink O Anciaux ML Derhy S Rougier P Lenoir C Attali P Etienne JP A randomized trial of hepatic arterial chemoembolization in patients with unresectable hepatocellular carcinoma J Hepatol 1990 11 181 184 10.1016/0168-8278(90)90110-D 2174933

[B13] Lau WY Lai EC The current role of radiofrequency ablation in the management of hepatocellular carcinoma: a systematic review Ann Surg 2009 249 20 25 10.1097/SLA.0b013e31818eec29 19106671

[B14] Kwon JH Bae SH Kim JY Choi BO Jang HS Jang JW Choi JY Yoon SK Chung KW Long-term effect of stereotactic body radiation therapy for primary hepatocellular carcinoma ineligible for local ablation therapy or surgical resection. Stereotactic radiotherapy for liver cancer BMC Cancer 2010 10 475 10.1186/1471-2407-10-475 20813065 PMC2940809

[B15] Cheng AL Kang YK Chen Z Tsao CJ Qin S Kim JS Luo R Feng J Ye S Yang TS Efficacy and safety of sorafenib in patients in the Asia-Pacific region with advanced hepatocellular carcinoma: a phase III randomised, double-blind, placebo-controlled trial Lancet Oncol 2009 10 25 34 10.1016/S1470-2045(08)70285-7 19095497

[B16] Seong J Han KH Park YN Nam SH Kim SH Keum WS Kim KS Lethal hepatic injury by combined treatment of radiation plus chemotherapy in rats with thioacetamide-induced liver cirrhosis Int J Radiat Oncol Biol Phys 2003 57 282 288 10.1016/S0360-3016(03)00540-6 12909244

[B17] Park HC Seong J Han KH Chon CY Moon YM Suh CO Dose-response relationship in local radiotherapy for hepatocellular carcinoma Int J Radiat Oncol Biol Phys 2002 54 150 155 12182985 10.1016/s0360-3016(02)02864-x

[B18] Milross CG Mason KA Hunter NR Terry NH Patel N Harada S Jibu T Seong J Milas L Enhanced radioresponse of paclitaxel-sensitive and -resistant tumours in vivo Eur J Cancer 1997 33 1299 1308 10.1016/S0959-8049(97)00107-X 9301459

[B19] Choy H Rodriguez FF Koester S Hilsenbeck S Von Hoff DD Investigation of taxol as a potential radiation sensitizer Cancer 1993 71 3774 3778 10.1002/1097-0142(19930601)71:11<3774::AID-CNCR2820711147>3.0.CO;2-0 8098270

[B20] Liu X Guo Y Li Y Jiang Y Chubb S Azuma A Huang P Matsuda A Hittelman W Plunkett W Molecular basis for G2 arrest induced by 2'-C-cyano-2'-deoxy-1-beta-D-arabino-pentofuranosylcytosine and consequences of checkpoint abrogation Cancer Res 2005 65 6874 6881 10.1158/0008-5472.CAN-05-0288 16061671

[B21] Watson C Miller DA Chin-Sinex H Losch A Hughes W Sweeney C Mendonca MS Suppression of NF-kappaB activity by parthenolide induces X-ray sensitivity through inhibition of split-dose repair in TP53 null prostate cancer cells Radiat Res 2009 171 389 396 10.1667/RR1394.1 19397439

[B22] Chen YJ Liao HF Tsai TH Wang SY Shiao MS Caffeic acid phenethyl ester preferentially sensitizes CT26 colorectal adenocarcinoma to ionizing radiation without affecting bone marrow radioresponse Int J Radiat Oncol Biol Phys 2005 63 1252 1261 10.1016/j.ijrobp.2005.08.001 16253780

[B23] Pawlik TM Keyomarsi K Role of cell cycle in mediating sensitivity to radiotherapy Int J Radiat Oncol Biol Phys 2004 59 928 942 10.1016/j.ijrobp.2004.03.005 15234026

[B24] Terasima T Tolmach LJ X-ray sensitivity and DNA synthesis in synchronous populations of HeLa cells Science 1963 140 490 492 10.1126/science.140.3566.490 13980636

[B25] Sinclair WK Cyclic x-ray responses in mammalian cells in vitro Radiat Res 1968 33 620 643 10.2307/3572419 4867897

[B26] Sinclair WK Morton RA X-ray sensitivity during the cell generation cycle of cultured Chinese hamster cells Radiat Res 1966 29 450 474 10.2307/3572025 5924188

[B27] Schmit TL Ahmad N Regulation of mitosis via mitotic kinases: new opportunities for cancer management Mol Cancer Ther 2007 6 1920 1931 10.1158/1535-7163.MCT-06-0781 17620424

